# Fracture Initiation Pressure as a Measure of Cemented Paste Backfill Strength

**DOI:** 10.1007/s42461-025-01257-6

**Published:** 2025-04-25

**Authors:** James A. Frimpong, Basel Ahmad Shabab, Rohit Pandey, Snehamoy Chatterjee, Gabriel Walton, Alexander S. Brand

**Affiliations:** 1 Mining and Minerals Engineering, Virginia Tech, 445 Old Turner St, Blacksburg, VA 24061 USA; 2 Geological and Mining Engineering and Sciences, Michigan Technological University, 630 Dow Environmental Sciences, 1400 Townsend Dr, Houghton, MI 49931 USA; 3 Geology and Geological Engineering, Colorado School of Mines, Berthoud Hall 311 A, 1516 Illinois St, Golden, CO 80401 USA; 4 Civil and Environmental Engineering, Virginia Tech, 305 A Patton Hall, Blacksburg, VA 24061 USA

**Keywords:** Cemented paste backfill, Fracture initiation pressure, Tensile strength prediction, Failure models

## Abstract

This laboratory-scale study presents the development and validation of a hydraulic fracturing technique to directly measure the tensile strength of cemented paste backfill (CPB), providing an alternative to traditional strength testing methods. Fracture initiation pressure (FIP) was used as the primary measure of CPB strength. Experimental results were compared with traditional benchmark measures such as uniaxial compressive strength (UCS), Brazilian tensile strength (BTS), and critical Mode-I fracture toughness (K_Ic_). Regression analysis of experimental results revealed a strong linear relationship between FIP and these benchmark strength measures, indicating that FIP can be used as a reliable predictor of CPB strength. However, traditional linear elastic failure models did not adequately explain the observed FIP values, as they significantly over-predicted the CPB tensile strength. To address this, the Point Stress (PS) model was applied, which provided a more accurate prediction of tensile strength, especially in cases involving small boreholes. The PS model explained observed effects of borehole size on the material’s response to hydraulic pressurization. This study confirms that hydraulic fracturing, interpreted through the PS model, is an effective method for determining CPB strength and provides a practical alternative measure to conventional testing methods.

## Introduction and Background

### Mining Challenges

The increasing demand for mineral resources and advances in extraction techniques led mining operations to target deeper ore bodies [1]. The deeper deposits are characterized by higher in situ stress levels and complex geological conditions, which pose significant geological challenges that must be carefully managed to ensure safe and efficient mining operations. Furthermore, mining of low-grade ore bodies produces large quantities of tailings waste, necessitating the development of environmentally responsible disposal strategies [2, 3]. Effective tailings management is critical to the sustainability of the mining industry [4].

Historically, disposal methods such as riverine and marine dumping have caused extensive and long-lasting environmental damage [5]. Surface tailings dams, a commonly used solution, also carry inherent risks, as demonstrated by catastrophic failures like the Aberfan disaster in 1966, where a tailings dam collapse tragically claimed 144 lives [6]. Such instances of dam failure have continued, where more recently, the Jagersfontein dam collapse at a diamond mine in South Africa in 2022 claimed the lives of 3 people, and injured over 300 [7]. As of 2020, the global rate of tailings dam failures as reported around 3.45 per year [8]. The uncontrolled release of tailings, whether through dam failure or surface seepage, poses significant threats to rivers, groundwater systems, and downstream communities. As mining continues to produce large volumes of waste, industry must explore and implement more sustainable tailings disposal alternatives.

### Backfill in Underground Mining

One major use of mine tailings, especially in underground metal mine operations is in the form of backfill to help provide structural support to mined-out areas. The practice of backfilling mined out areas has been common practice in such operations, which can be dated back to the 1500’s to mines in Mexico [9]. Although historical fills included timber, trash and mine waste, modern backfills are heavily engineered. The geotechnical properties of the orebody and host rock play a critical role in determining the suitability of backfill operations. Specifically, mining methods such as vein-type mining or stope-type mining, which often occur in weak host rock environments, can benefit from the use of backfill to stabilize surrounding rock formation and prevent collapses. In weak host rock conditions, backfill helps maintain ground stability and prevents excessive deformation of the surrounding rock mass, making it an essential part of mining in such settings. At present, there exist five major types of backfill commonly used in mining operations: cemented paste backfill (CPB), cemented hydraulic fill (HF), cemented aggregate fill (CAF), cemented rock fill (CRF), and waste rock fill (WRF). The choice of backfill type depends on various factors, including the geomechanical properties of the surrounding rock, the size and geometry of the stope, water conditions, and the availability of materials. Additionally, the ability to provide long-term stability, minimize subsidence, and reduce environmental impacts are key considerations when selecting the appropriate backfill method for a specific mining operation. A number of comprehensive reviews provide detailed discussions and guidance on the various types of backfill and the criteria for selecting the most suitable option for specific mining conditions [10–13].

Due to its low operational costs and the ability to incorporate large quantities of waste tailings, mining operations are increasingly adopting CPB as their primary backfill method [14], which is the focus of the work presented in this study. Notable examples include the mines in the Carlin Trend, such as Goldstrike Meikle and the Ren extension, which are in advanced stages of enhancing their pastefill capacities [15]. Future projects like South32’s Hermosa mine [16] and Hindustan Copper’s Malanjkhand mine [17] are also planning to implement CPB-based operations.

**Fig. 1:**
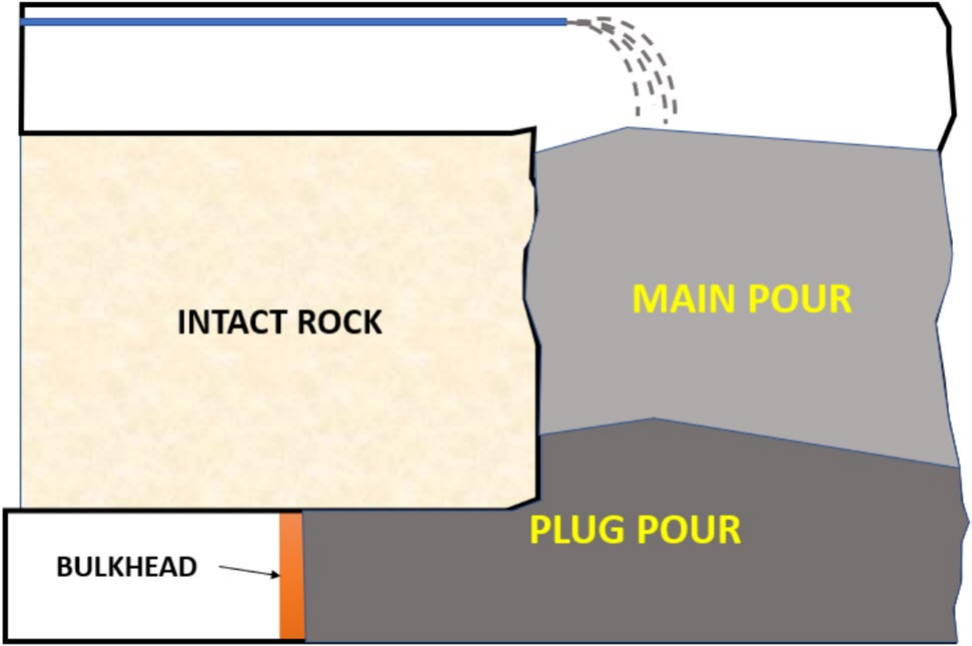
Typical stope layout during paste pour showing plug and main pours held in place by a bulkhead

The strength and performance of the backfill in in situ conditions are crucial when planning mining operations. Ensuring it meets the required strength is essential for maintaining mine integrity and supporting safe, efficient practices. Specifically, for CPB, determination of its strength is crucial at various stages, including, early and late stage. Early-stage strength is vital to ensure that the poured paste backfill can support its own weight and does not depend on the engineered bulkhead for stability. Personnel and equipment are only permitted near the pour site once the CPB is able support itself, enhancing safety in the event of bulkhead failure—a scenario that has been documented multiple times [18, 19], applicable for both stope and drift pours. Another critical scenario for early-stage strength occurs when mining operations plan to fill stopes continuously, maintaining the bulkhead’s integrity without resorting to the traditional two-step approach: plug and main pour. In the traditional approach, the first plug pour is applied to a few feet above the stope brow to limit the pressure on the bulkhead, as seen in Fig. 1. The main pour only begins once the initial plug achieves sufficient frictional strength to prevent additional load on the bulkhead, which could take several days. During this period, any mining activity that risks being affected by potential bulkhead failure, leading to engulfment, is prohibited, thus impacting mine productivity. Industry practices typically wait for the plug pours to reach a strength of roughly 22 psi before adding the weight of the main pour [20]. However, as covered in subsequent Sect. 1.3, current standard industry practices do not involve measurement of in situ strength of a backfill stope. On the other hand, assessment of late-stage CPB strength is essential for providing the necessary structural support to prevent uncontrolled ground failures. Ensuring CPB has sufficient late-stage strength also enables mining operations to safely plan for mining areas below or adjacent to the paste pour [21].

### Current practices to measure CPB strength

**Fig. 2:**
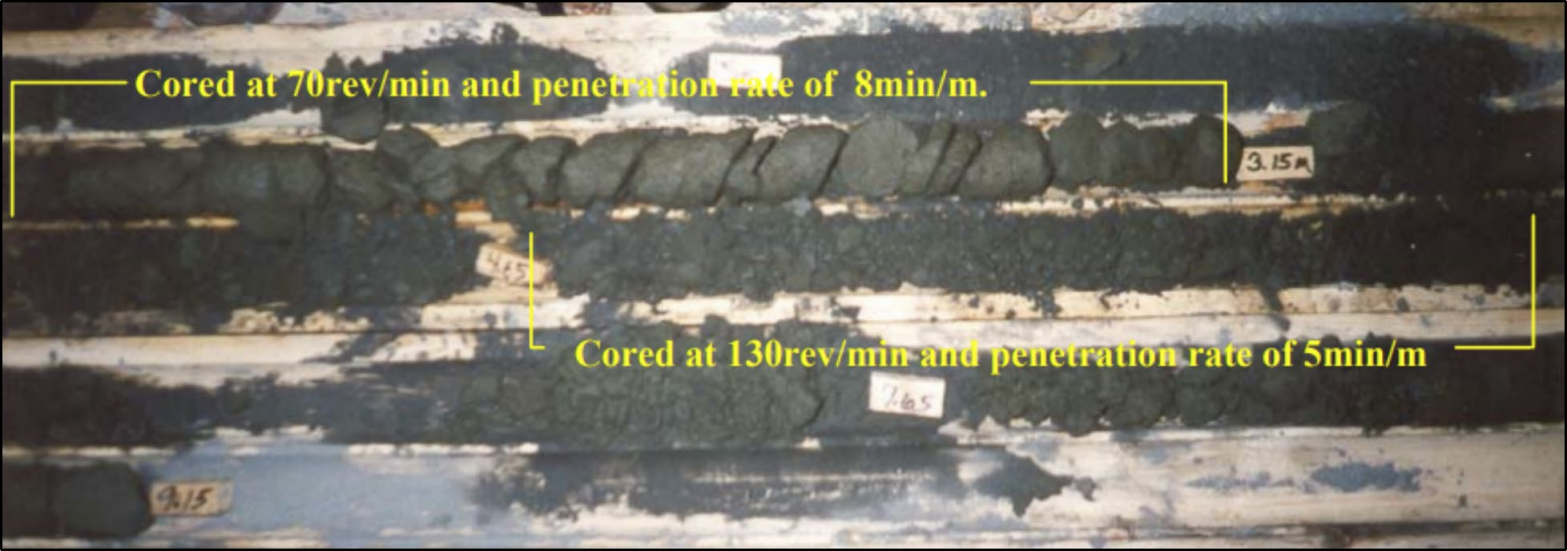
Condition of retrieved CPB core samples obtained (NQ-3 size core barrel) from an in situ location [23]

Current operational practices to measure CPB strength require samples to be cast in cylinders at the paste plant in curing rooms. The cylindrical samples are then used to measure the uniaxial compressive strength at fixed intervals to estimate the strength of the CPB. Such practices are not representative of actual in situ conditions and yield unreliable results (coefficient of variation between 15 to 60%, compared to 3 to 6% in the concrete industry) [22]. In cases where in situ strength determination becomes critical, the use of cone penetration tests, self-boring pressuremeters, or coring from paste locations is undertaken [23–27]. These methods are associated with both operational and technical challenges. In situ pastefill coring is commonly resorted to when mine operations require reliable measures of strength. However, given the relatively low strength of CPB, the material is prone to failure during the coring process, making core-retrieval very challenging. This issue is illustrated in Fig. 2, where none of the retrieved core samples during a field scale study at the Golden Giant Mine in Northern Ontario were usable [23]. After failed initial attempts at retrieving cores, the researchers ultimately resorted to carving out a block from the top of a stope to then obtain core samples. Alternate methods such as the cone penetration tests have primarily been developed for geotechnical investigation of soils and require monitoring of various parameters such as cone tip resistance, sleeve friction, dynamic pore water pressure, temperature, and cone inclination [24]. These values are then analyzed empirically to arrive at relevant geotechnical data [26]. Evidently, reliable data from such tests require extensive calibration arrangements and sound technical interpretation, which limits its applicability in mining environments.

Recently, electrical conductivity (EC) method has been proposed for measuring the strength of CPB [28]. This method capitalizes on the fact that during the initial stages of hydration, there is an increased concentration and mobility of ions such as OH^−^, Na^+^, K^+^ and SO_4_^−^ within the pore solution. As CPB cures and its strength develops, the formation of calcium silicate hydrate (CSH) bonds becomes the primary factor in strengthening the material. The formation of CSH consumes ions in solution, leading to a reduction in the mobility of ions and consequently, a decrease in EC. Since this decrease in ion concentration (and EC) directly correlates with the formation of CSH bonds, EC has shown potential as a reliable indicator of CPB strength. Jafari et al. [28] investigated this relationship, reporting an initial increase in EC within the first few hours of CPB mixing as ions are formed, followed by an asymptotic decrease in EC as hydration progresses. Uniaxial compressive strength measurements taken during early curing stages (1, 2, 3, and 7 days) demonstrated a strong logarithmic correlation with EC values, which can be leveraged by mine operators, especially to continuously monitor the early-age strength gain in CPB samples at in situ locations. However, this method relies on an indirect assessment of CPB strength and is currently not suitable for use in locations that were not instrumented before backfilling.

Thus, the primary objective of the work presented here is to develop a hydraulic fracturing technique aimed at directly measuring the strength of CPB. Hydraulic fracturing has been widely used in related industries to especially measure the in situ stress state of geologic formations [29]. Additionally, hydraulic methods have also been used to measure a number of geomechanical properties of rocks and geomaterials [30–32]. However, the use of hydraulic fracturing to measure the strength of CPB has not been reported. Given the novelty in the application, the initial focus as presented here will be to experimentally establish the validity of the proposed technique as a measure of geomechanical strength of CPB, and to characterize the late-stage strength of CPB (3 + days). Additionally, a theoretical analysis of relevant failure models is presented to support the use of hydraulic fracturing as an effective measure of CPB strength.

### Fundamental Theories Relating Fracture Initiation Pressure and Material Strength

Hydraulic fracturing involves creating tensile fractures in rock by injecting pressurized fluid into drilled and cased boreholes, allowing the fluid to transfer pressure onto the borehole walls either directly or indirectly [33]. As injection continues, hydraulic pressure increases until it reaches a peak, causing tensile cracks to propagate, after which the pressure drops. The fracture initiation pressure (FIP), which is the maximum pressure at which these fractures are initiated, is of primary importance in the work presented here. Given the nature of failure, FIP strongly correlates with the tensile strength of the geomaterial [34, 35]. This relationship enables potential application of hydraulic fracturing to provide a measure of CPB strength. This testing methodology potentially offers a practical and feasible solution to enable mine operators to measure CPB strength, even in challenging in situ locations that have until now proven difficult and resource intensive to evaluate.

The relationship between FIP, tensile strength and the state of stress has been investigated by numerous researchers. Most notably, the seminal work of Hubbert and Willis [34] provides insight into the use of fracturing to calculate either the in situ stress or the formation tensile strength (prior knowledge of one is required to determine the other). Their model, also referred to as the Linear Elastic (LE) model assumes linear, elastic, homogeneous, isotropic and continuous impermeable rock. The LE formulation is expressed as the following Eq. 1:

1 $$FIP= {\sigma }_{T}+3{\sigma }_{1}- {\sigma }_{3}- {P}_{0}$$

where ${\sigma }_{T}$ is the tensile strength, ${\sigma }_{1}$ is the applied major principal stress under confinement, and ${\sigma }_{3}$ is the minor principal stress, with ${P}_{0}$ being the initial pore pressure of the fluid in the material. For unconfined samples, tested in the laboratory with no pore-fluids present, similar to the samples tested in the work presented here, the LE formulation further reduces to Eq. 2, where the FIP will effectively measure the tensile strength of the geomaterial, in our CPB.

2 $$FIP= {\sigma }_{T}$$

An alternate approach to understand the dependence of FIP on the tensile strength, as derived by in the LE model is to understand the failure theories associated with internally pressurized thick wall vessels. Thick-walled cylinders, by convention, are considered to be cylinders where the ratio of the wall thickness to the internal diameter is greater than, or at minimum equal to 0.05 [36]. Failure of internally pressurized thick-walled cylinders has been widely reported, and readers are directed to the comprehensive work complied by Vullo [36] for an in-depth analysis of stress distributions and failure mechanisms, which has been reported for a variety of boundary conditions. Pertinent to the work reported here, the instance of internally pressurized (zero external confinement) systems will be considered.

**Fig. 3:**
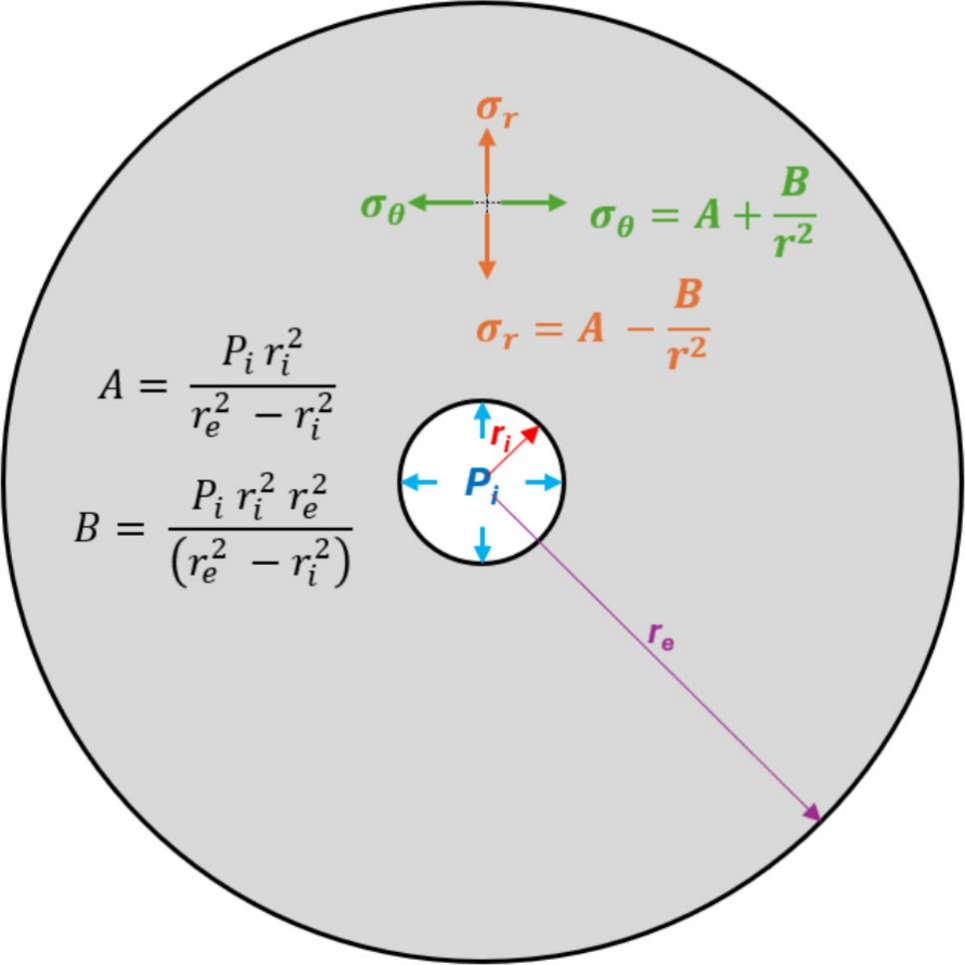
Cross-section of a pressurized thick-walled cylinder with relevant expressions for radial and hoop stress induced by internal pressurization

Figure 3 presents a basic cross-section of an internally pressurized thick-walled cylinder of radius *r*_*e*_, where fluid pressure *P*_*i*_ is applied to the confines of the borehole of radius *r*_*i*_. Since the system is expected to operate in a plane-strain condition, a two-dimensional analysis is appropriate. The borehole pressurization results in the formation of radial stress ${\sigma }_{r}$, and hoop-stress ${\sigma }_{\theta }$ in the wall of the cylinder, expressed as the following equations:

3 $${\sigma }_{\theta }=A+\frac{B}{{r}^{2}}$$

4 $${\sigma }_{r}=A -\frac{B}{{r}^{2}}$$

where $r$ is any radial distance along the cylinder wall, *A* and *B* are functions of the radius of ${r}_{i}$, ${r}_{e}$, and ${P}_{i}$, and is expressed as the following equations:

5 $$A= \frac{{P}_{i }{r}_{i}^{2}}{({r}_{e}^{2} -{r}_{i}^{2})}$$

6 $$B= \frac{{P}_{i}{ r}_{i}^{2}{ r}_{e}^{2}}{\left({r}_{e}^{2} -{r}_{i}^{2}\right)}$$

Continued internal pressurization will result in eventual failure of the cylinder, wherein tensile cracks will be generated in the cylinder wall. The strength theories that guide this failure process assume that the cylinder will fail when the equivalent stress in the borehole wall (${\sigma }_{e}$) will equal and then exceed the tensile strength of the cylinder. Numerous theories exist that aim at relating the geometric dimensions of the thick-walled cylinder, the size of the borehole, the tensile strength of the material and the pressure at which failure would occur [36]. This failure pressure is akin to FIP as defined previously. The commonly used failure theories include the maximum stress theory (${\sigma }_{\text{max}}$), maximum shear theory (${\tau }_{\text{max}}$), and the Energy Distortion theory (${ED}_{\text{max}}$). For plane-strain conditions, Table 1 provides the failure conditions in accordance with each of these theories. Hubbert and Willis’s [34] LE model is effectively a specific case of the ${\sigma }_{\text{max}}$ failure theory.

**Table 1:** Theories associated with the failure of internally pressurized thick-walled pressure vessels

Strength theory	Failure condition	Equation number	
$\sigma_{max}$	$\sigma_e\;=\;\sigma_t\;=\;A\;+\;\frac B{r^2}$	Eq. 7	
$\tau_{max}$	$\sigma_e\;=\;\sigma_t\;=\;\frac{2B}{r^2}$	Eq. 8	
$ED_{max}$	$\sigma_e\;=\;\sigma_t\;=\;\sqrt{A^2\;+\;3\;\frac{B^2}{r^4}}$	Eq. 9	

Based on the formulations presented by the LE model and the failure theories derived by analysis of thick-walled cylinders, knowledge of the geometric dimensions of the system along with the measured fluid pressure at which failure occurs (FIP) should enable direct calculation of the tensile strength of the material. This forms the basic scientific premise of the approach to using FIP as a measure of CPB tensile strength. To the best of the authors’ knowledge, no previous studies have evaluated the hydraulic fracturing mechanism in CPB. Additionally, the validity of the cited failure criteria and the established correlations between FIP and traditional benchmark measures of strength have not been examined.

Thus, this study focuses on developing a methodology for laboratory-scale hydraulic fracturing of CPB samples under unconfined conditions and establishing relationships between the measured FIP and traditional benchmark measures of strength. The experimental data will be utilized to evaluate the effectiveness of standard failure criteria in predicting hydraulic fracturing behavior in CPB. Specifically, the objectives include establishing an experimental protocol for hydraulic fracturing of CPB, evaluating the relationship between FIP and traditional CPB strength measures—uniaxial compressive strength (UCS), Brazilian tensile strength (BTS), and mode-I fracture toughness (K_Ic_), and providing a theoretical framework to enable the use of hydraulic fracturing as a tool for assessing CPB strength.

## Experimental Plan

**Fig. 4:**
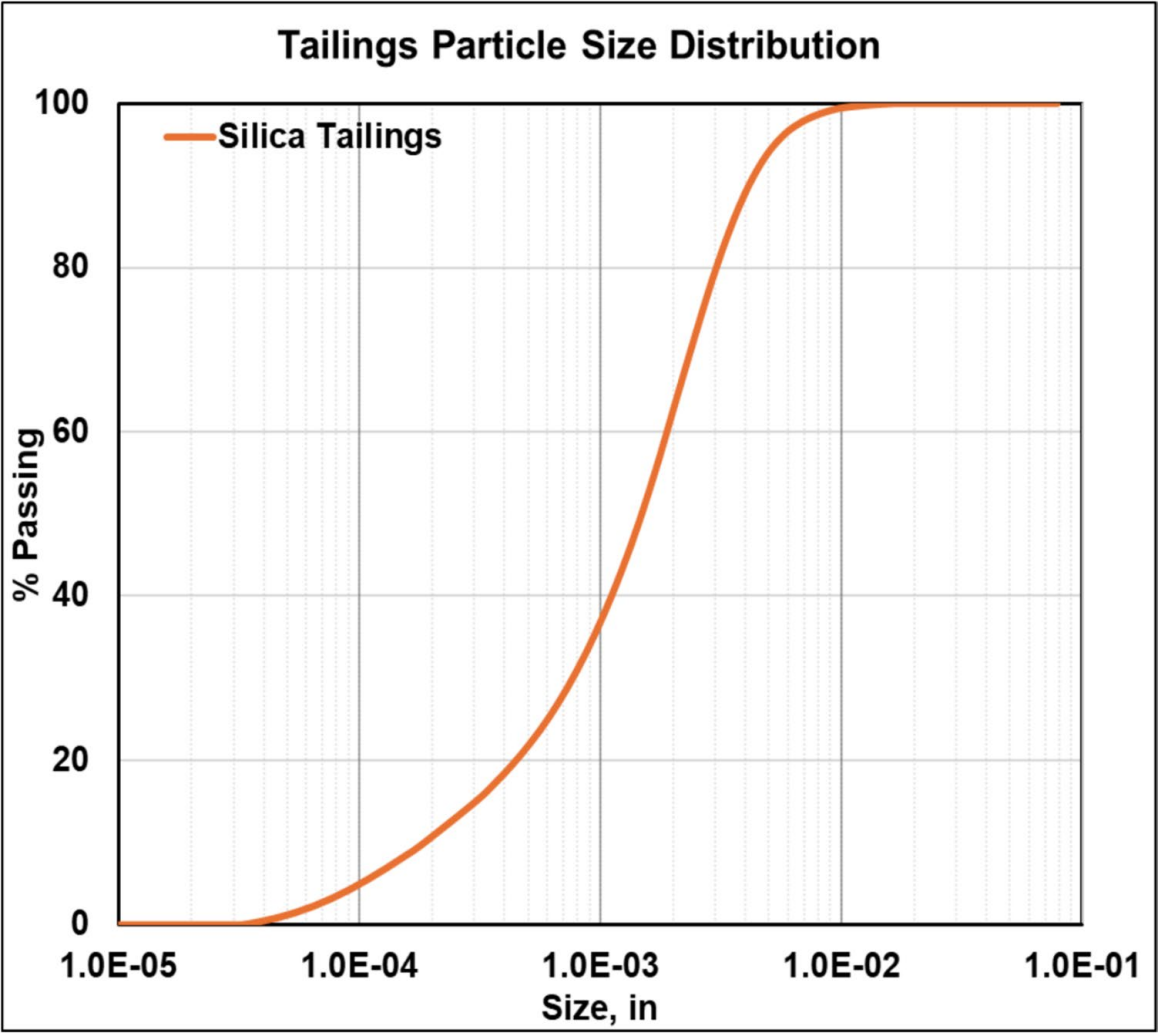
Particle size distribution of the silica tailings used in CPB preparation

**Fig. 5:**
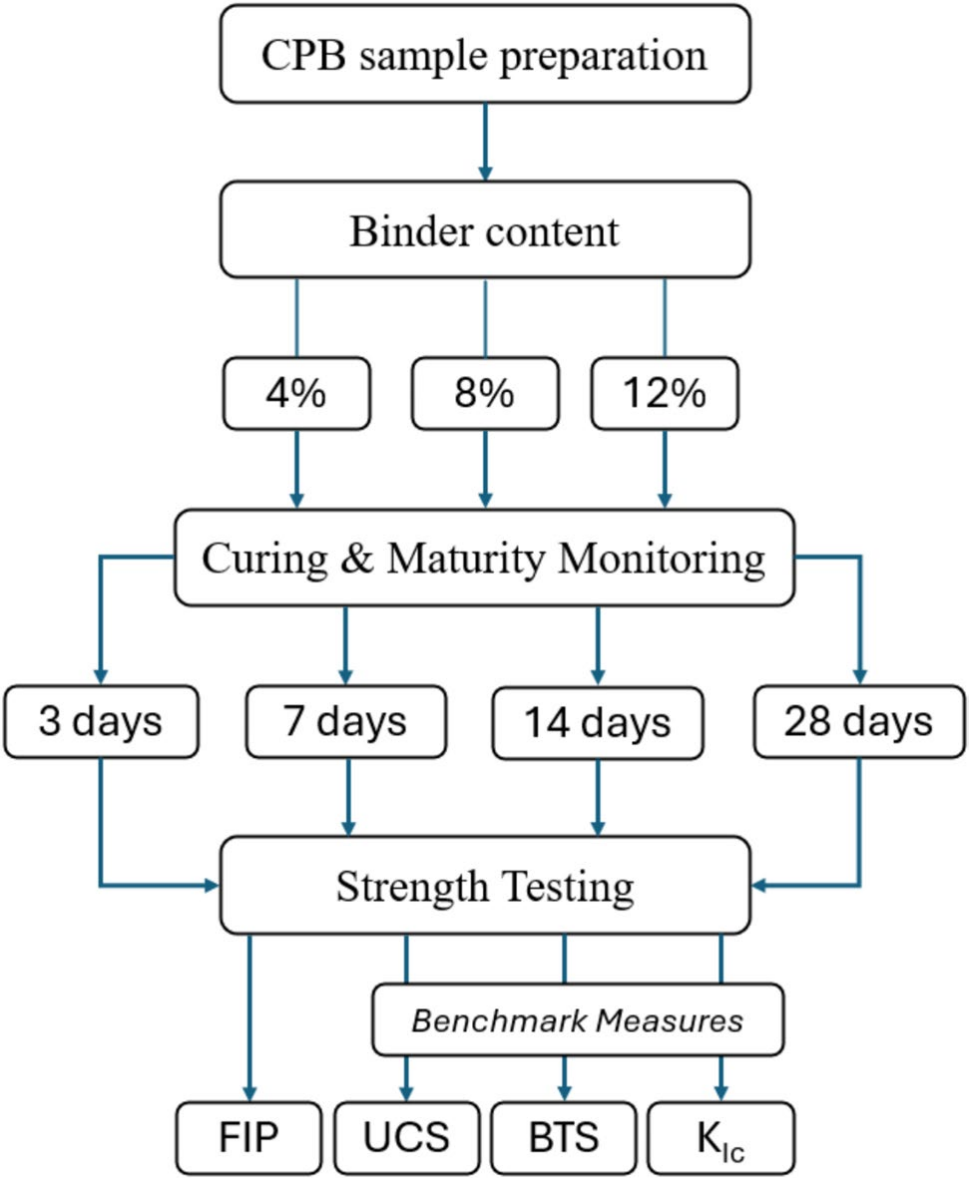
Experimental plan followed in the proposed research

The experimental design of this research involved using CPB samples, prepared using silica fines with a particle size distribution which is representative of actual mine tailings. The particle size distribution for the silica tailings is presented in Fig. 4. Based on size classification introduced by Sivakugan et al. [37], the silica tailings are well suited for CPB preparation. The CPB samples were prepared with varying binder contents of 4%, 8%, and 12% (by weight of total solids) and tested at different curing periods of 3, 7, 14, and 28 days. For each combination of binder content and curing time, samples were subjected to a series of tests, including hydraulic fracturing to measure FIP, UCS, BTS, and K_Ic_ tests. The relationships among these parameters were analyzed to assess the validity of using hydraulic fracturing as an indicator of CPB geomechanical strength. Tests were performed in triplicates to ensure the reliability and consistency of the results. Figure 5 presents a schematic of the experimental plan for the proposed research.

### Experimental Method

Specifics of the procedure followed to enable preparation of CPB and the measurement of the various properties are provided in the following subsections.

#### Preparation of CPB Mixture

The CPB samples were prepared using silica fine tailings, Type I Portland cement, and tap water. The solids content of the CPB mixtures was maintained at 75% by weight, while the binder content was varied at 4%, 8%, and 12% by weight of total solids. The mixtures were prepared by thoroughly mixing the materials in a mixer for 15 min at a constant speed to ensure homogeneity. After mixing, the CPB was poured into molds of various shapes and sizes, depending on the specific test requirements. For fracturing tests and UCS tests, 2″ by 4″ cylindrical molds were used, while 2″ diameter discs were prepared for BTS tests and fracture toughness tests. All the prepared CPB samples were seal cured at room temperature for the designated curing times of 3 days, 7 days, 14 days, and 28 days. Additionally, to prevent moisture loss during the curing process, the samples were covered and maintained in a fog-room environment, ensuring consistent curing conditions throughout the curing period.

#### CPB Maturity Monitoring

Maturity monitoring of the CPB samples was conducted to investigate the evolution of the CPB properties during the curing process. In this study, maturity monitoring was performed using the TEROS12 sensor, manufactured by the METER Group. This type of sensor is primarily designed for soil monitoring but has found a unique application in monitoring CPB maturity [28]. The sensors enable measurement of EC of CPB. The sensors were connected to a ZL6 data logger, which collected and stored the sensor data at 15-min intervals throughout the curing process. Since the TEROS12 sensors have a minimum volume requirement, the monitoring was conducted in 6″ cubic samples.

#### Benchmark Strength Measurement

The strength of CPB was assessed using three benchmark tests: UCS, BTS, and K_Ic_. UCS tests were conducted according to ASTM C39/C39M-21 [38] using an INSTRON 5982 testing machine with of 22 kips capacity, applying a controlled displacement loading rate of 0.0039 in/min until failure. The maximum load before failure was recorded and used to calculate UCS. BTS tests followed ASTM C496/C496M-17 [39], using the same machine with disc samples loaded diametrically at a rate of 0.008 in/min. BTS was calculated based on the maximum load and sample dimensions. The K_Ic_ was measured using the semi-circular bend (SCB) method developed by Chong and Kuruppu [40]. Semi-circular CPB samples with a vertical notch of 0.5″ were loaded at a rate of 0.008 in/min until failure, and K_Ic_ was calculated using the recorded load and sample dimensions.

#### Fracture Initiation Pressure Measurement

**Fig. 6:**
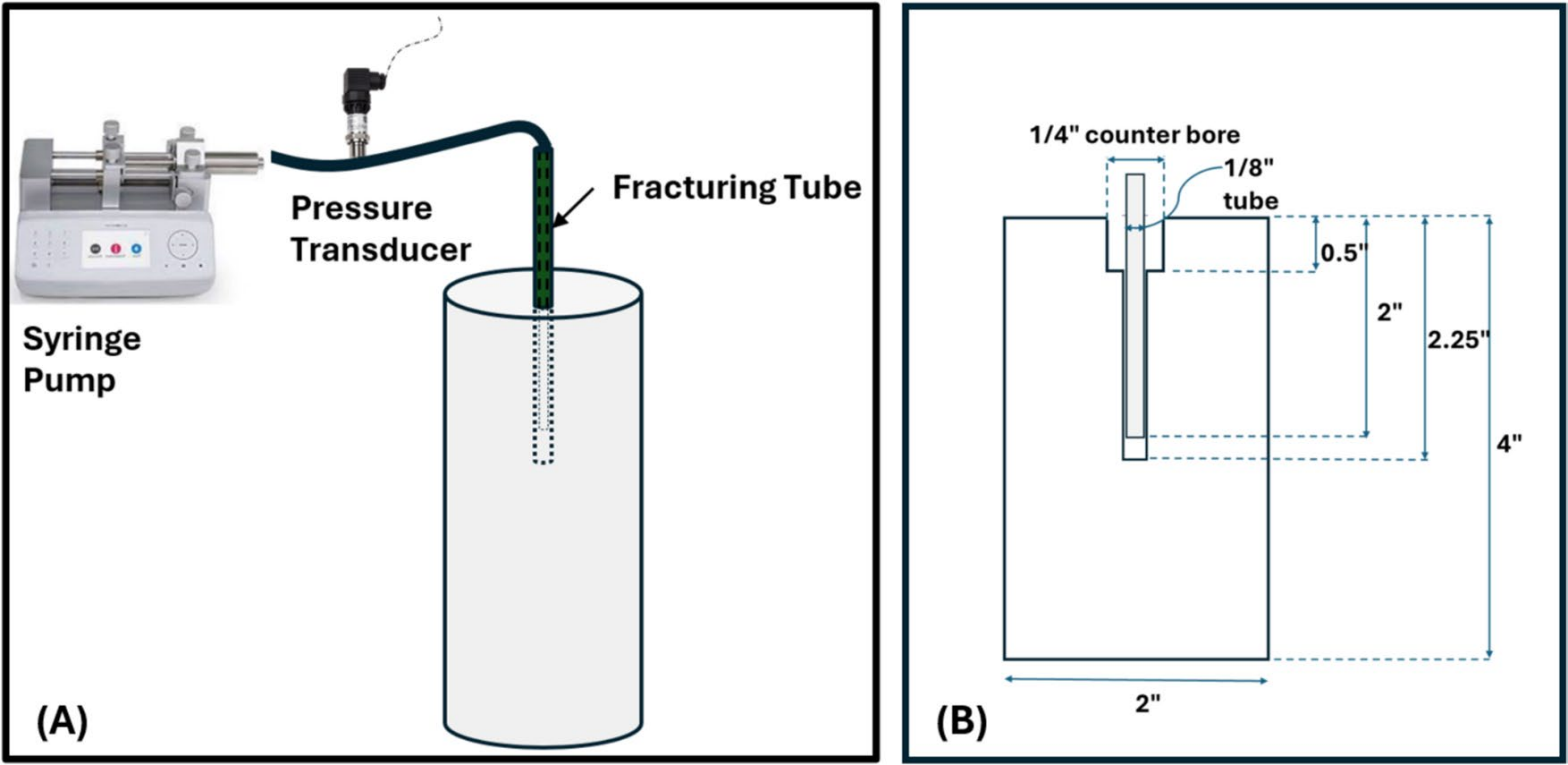
**A** Schematic of the fracture initiation setup. **B** Dimensions of the sample and the borehole

A fracture initiation system was developed to conduct hydraulic injection tests on the CPB samples. As shown in Fig. 6A, the system consisted of a flow-controlled syringe pump connected to a pressure transducer for real-time pressure monitoring. The calibrated pressure transducer had a maximum pressure rating of 3000 psi, with a maximum full-scale error of 0.2%. The syringe pump was capable of delivering a constant flow rate of the fracturing fluid, which in this study was AW32 hydraulic oil. As seen in Fig. 6B, the samples were 2″ by 4″ cylinders, and the fracturing tube was 1/8″ diameter stainless steel tube. When the CPB sample was ready to be tested, a drill bit of 1/8″ diameter was used to create a hole through the center of the sample to a depth of 2.25″. The fracturing tube was then inserted into the hole to a depth of 2″, leaving a 0.25″ open hole at the bottom. A drill bit of 1/4″ diameter was used to create a counter bore through the center of the drilled hole to a depth of 0.5″, and quick-setting high strength epoxy glue was applied to keep the tube in place. As the syringe pump injected the hydraulic oil at a constant flow rate (0.244 cu.in/min), the pressure within the sample increased until it reached a maximum value, i.e., FIP. At that point, the sample fractured, and the pressure rapidly decreased.

## Results

The experimental results obtained from testing CPB samples with different binder conditions and curing times are presented in this section.

### Maturity Monitoring

**Fig. 7:**
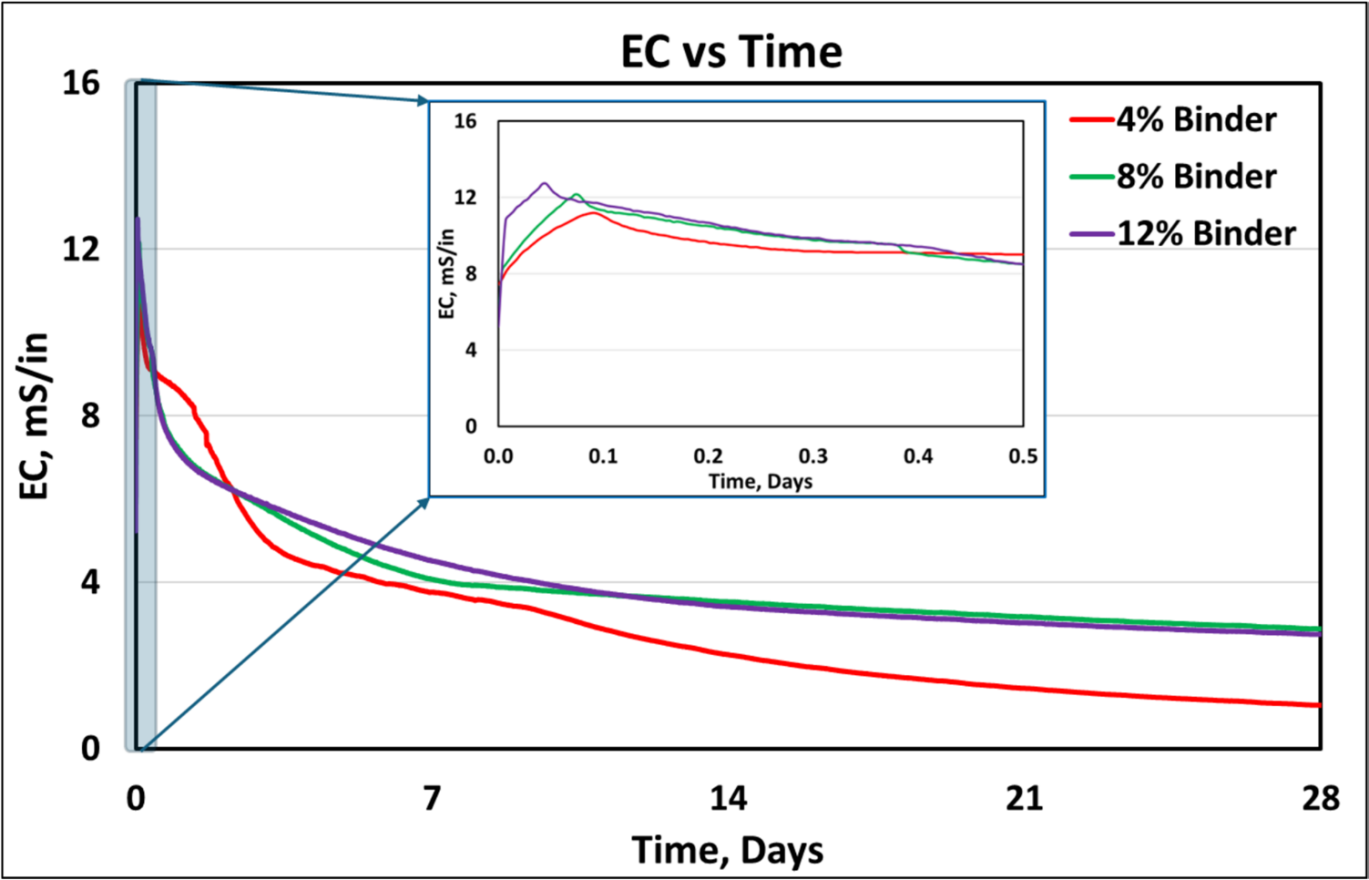
Development of electrical conductivity of CPB samples. The insert figure plots the data for the initial 12 h of electrical conductivity development in the CPB samples

The EC of the CPB samples was monitored over the 28 days of curing and is presented in Fig. 7. The insert in Fig. 7 presents the EC evolution in the first 12 h of curing. As expected, in the first few hours of curing, the EC of the CPB sample increased due to the dissolution of ions into the solution [28]. The highest EC measured for the 4%, 8%, and 12% binder samples were 12.72 mS/in, 12.13 mS/in, and 11.39 mS/in, respectively. The peak EC was developed first for the sample with the highest binder content, i.e., 12%, followed by CPB with 8% binder, and finally the CPB with 4% binder content. Since a higher binder content allows for a higher concentration of mobile ions, the early onset of peak EC for CPB samples having higher binder content is as expected [28]. As the C-S–H bonds start forming, the EC for all three CPB mixtures begin decreasing. This aspect is clearly evident in Fig. 7, consistent with results reported in a prior study [28]. At the end of 28 days of cure, the 4% CPB sample had the lowest EC, at 1.05 mS/in. The ECs of the 8% and 12% CPB samples at the end of 28 days of cure were fairly similar at approximately 2.75 mS/cm.

### Hydraulic Injection and FIP

**Fig. 8:**
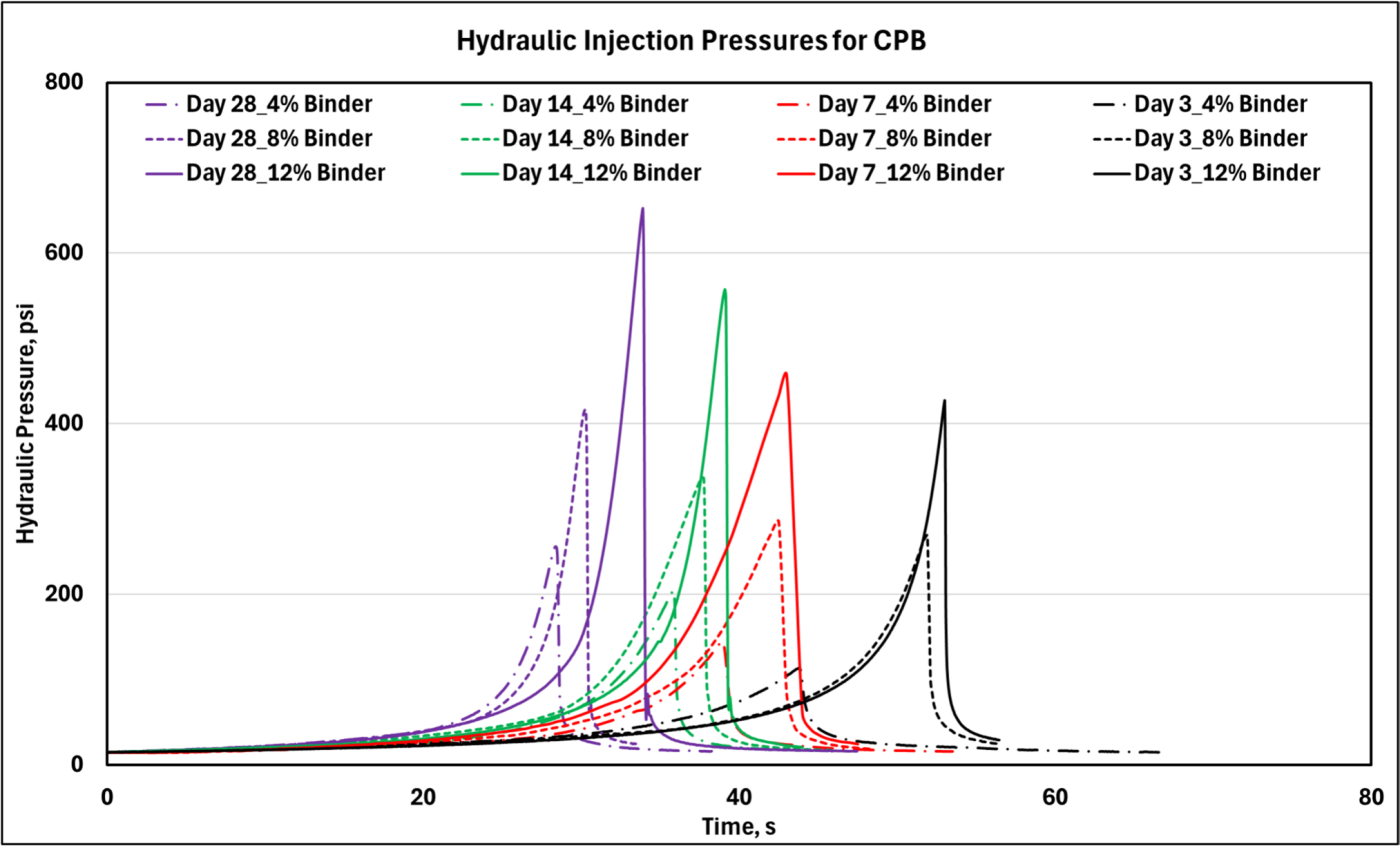
Hydraulic injection pressures obtained during fracturing of CPB samples at various binder content and cure periods. Note: Experiments were completed on triplicate samples at minimum. Pressure curves obtained for only a single representative sample are presented here. The FIP measures have been reported along with the corresponding standard deviation values in the subsequent work

The mean FIPs and corresponding standard deviation values that were obtained for the CPB samples at the multiple cure periods are presented in Table 2. Note that the experiments were repeated using three distinct samples for each unique condition (of binder content and cure period). Only a single hydraulic injection pressure curve obtained for each binder content and cure period combination is shown in Fig. 8.

**Table 2:** Mean and standard deviation (within parenthesis) of the fracture initiation pressure values obtained for CPB samples

Day	Binder (%)	FIP (psi)
3	**4**	117.6 (±37.8)
	**8**	261.0 (±27.4)
	**12**	411.7 (±66.6)
7	**4**	142.4 (±21.3)
	**8**	264.8 (±28.5)
	**12**	444.1 (±39.7)
14	**4**	205.7 (±12.8)
	**8**	338.3 (±11.5)
	**12**	565.8 (±54.8)
28	**4**	250.5 (±34.1)
	**8**	415.1 (±1.5)
	**12**	639.3 (±27.6)

The hydraulic pressure curves shown in Fig. 8 indicates that the FIP, defined as the maximum pressure at which the CPB sample fractured under hydraulic injection, increased with both higher binder content and longer curing times. This trend is consistent with expectations, as higher binder content leads to the formation of more CSH bonds, resulting in stronger samples [28]. Similarly, extended curing time allow for further bond development, enhancing the overall strength and increasing FIP. Overall, the results qualitatively support the primary hypothesis that stronger samples fracture at higher injection pressures. The accuracy of FIP as a quantitative indicator of material strength will be evaluated in Sect. 3.3 through regression analysis.

Further analysis of the hydraulic pressure curve in Fig. 8 suggests that the onset of FIP was delayed in all the samples at the 3-day cure timepoint. As curing progressed, the onset of FIP occurred more rapidly. As a CPB sample cures, a greater number of CSH bonds form over time, which results in reduction of sample porosity [41]. As porosity decreases, the leak-off effect during fluid injection is reduced, leading to lower volumes of hydraulic fluid, AW32 hydraulic oil in this case, seeping into the CPB samples from the borehole wall. This reduction allows for quicker pressurization of the oil, which is injected at a constant flow rate of 0.244 cu.in/min. The leak-off effect, however, did not significantly impact the actual FIP during fracturing, as will be demonstrated in Sect. 3.3.

### Measures of Strength

**Fig. 9:**
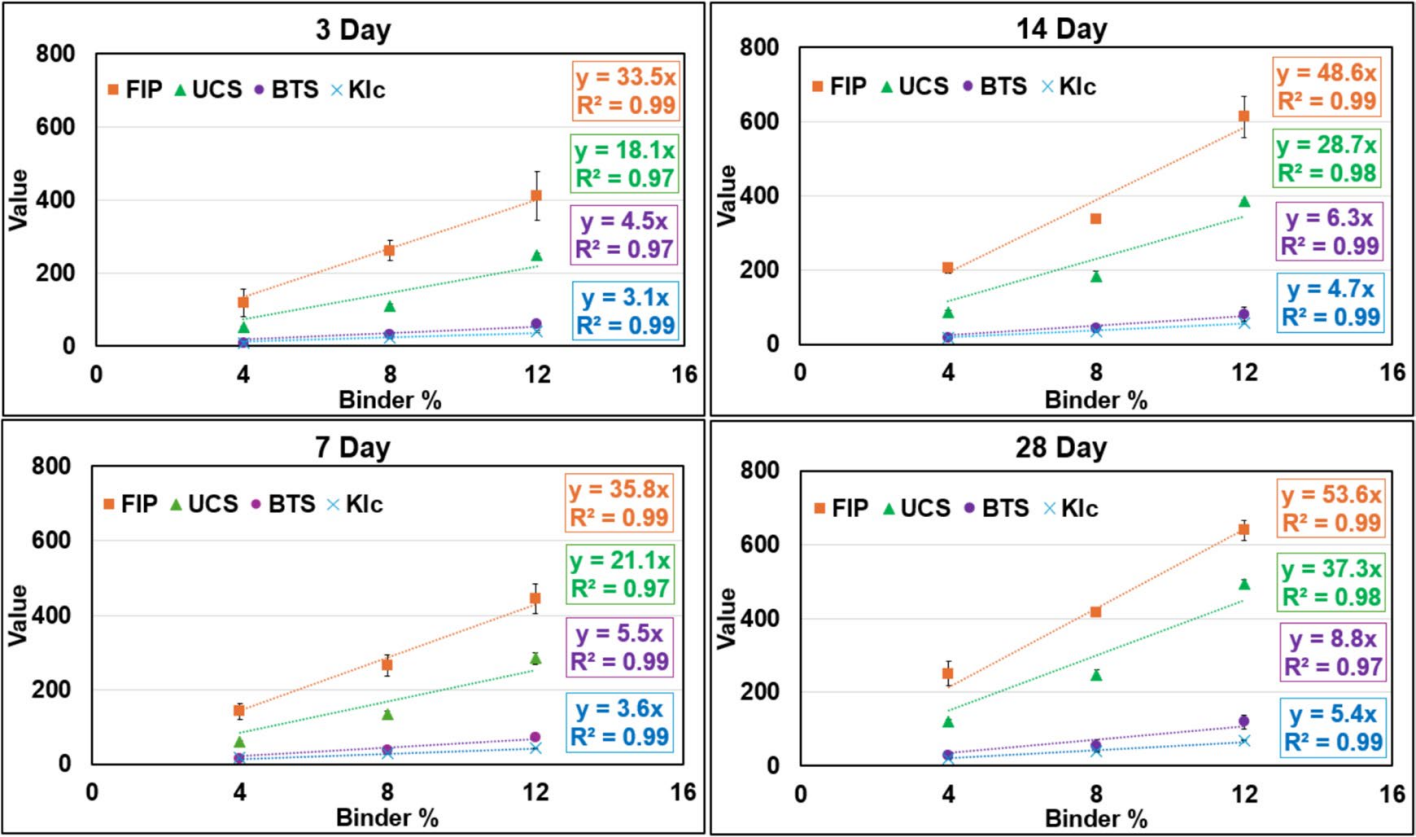
Strength of CPB with respect to binder content and curing time, as measured by benchmarked measures of strength and the fracture initiation pressure. The values for FIP, UCS and BTS along the *Y* axis is in psi, and K_Ic_ is in psi√in

Table 3 presents the mean values of these parameters and FIP, along with their corresponding standard deviations. Figure 9 provides a graphical visualization of all measures of strength as a function of binder content, with individual figures for each curing time point. As expected, increasing binder content and curing time results in enhanced strength of CPB samples, a trend consistently observed across all measures of strength.

**Table 3:** Mean and standard deviation (within parenthesis) values of the measures of benchmark strength measures and FIP for CPB

Day	Binder %	UCS (psi)	BTS (psi)	K_Ic_ (psi√in)	FIP (psi)
3	**4**	51.4 (± 3.2)	9.4 (± 0.4)	9.3 (± 1.9)	117.6 (± 37.8)
	**8**	110.0 (± 4.6)	30.3 (± 3.5)	22.8 (± 2.6)	261.0 (± 27.4)
	**12**	248.9 (± 5.2)	59.8 (± 5.0)	39.4 (± 2.3)	411.7 (± 66.6)
7	**4**	60.4 (± 3.4)	15.4 (± 2.4)	16.7 (± 2.6)	142.4 (± 21.3)
	**8**	135.6 (± 8.3)	38.9 (± 4.1)	28.5 (± 2.8)	264.8 (± 28.5)
	**12**	283.6 (± 16.5)	71.8 (± 9.5)	42.0 (± 0.4)	444.1 (± 39.7)
14	**4**	85.3 (± 6.2)	18.8 (± 4.1)	18.8 (± 0.7)	205.7 (± 12.8)
	**8**	184.2 (± 12.1)	44.8 (± 2.7)	36.2 (± 3.0)	338.3 (± 11.5)
	**12**	385.3 (± 3.8)	81.2 (± 18.2)	58.2 (± 2.1)	565.8 (± 54.8)
28	**4**	120.0 (± 6.2)	27.1 (± 4.6)	18.7^*^ (± 0.3)	250.5 (± 34.1)
	**8**	245.6 (± 14.0)	54.9 (± 17.6)	42.6 (± 2.1)	415.1 (± 1.5)
	**12**	493.6 (± 10.8)	119.4 (± 19.2)	69.5 (± 1.4)	639.3 (± 27.6)

^*^Note: The mean K_Ic_ value measured for 28-day cure at 4% binder is an outlier. Several samples failed prior to expected value, possibly due to weakening during sample handling and preparation

Regression analysis of strength measures versus binder content offers additional insight into the evolution of CPB strength. Data obtained at all four curing periods, as shown in Fig. 9, clearly indicates that CPB strength increases approximately linearly with increased binder content. The R^2^ values across all datasets were consistently high (> 0.97), confirming the robustness of the linear relationship. The linear nature of the relationship between UCS and binder content has also been reported in previous studies [28, 42]. Notably, at all curing periods, the regression slope of the strength vs. binder content relationship was greatest for FIP, followed by UCS, BTS, and K_Ic_. This suggests that FIP, as measured in our experimental setup, not only provides a reliable measure of CPB strength but is also a sensitive metric for interpreting CPB strength.

Furthermore, the slope of the regression curves plotting the relationship between strength and binder content increased with longer curing durations. This trend was consistent across all measures of strength—UCS, BTS, K_Ic_, and FIP. It implies that strength gain in CPB samples with higher binder content is greater over time compared to samples with lower binder content. The higher binder content facilitates the formation of CSH bonds over time, resulting in continued strength gain and explaining the observed trend. Analyzing the slope values presented in Fig. 9, the average slopes at the 7-day, 14-day, and 28-day marks were approximately 1.15, 1.49, and 1.84 times higher than the slope at Day 3. Previous studies [28, 42–45] have similarly demonstrated that CPB strength increases with higher binder content due to enhanced CSH bond formation. Optimal binder contents, however, depend on mine-specific conditions including geomechanical requirements, curing time, and tailings characteristics, as extensively discussed in prior works [10–13].

A key observation here is that the measured fracture initiation pressure consistently exceeds the tensile strength of the CPB, contradicting the theoretical framework of the linear elastic failure models discussed in Sect. 1.4. While higher FIP values are advantageous for providing greater resolution in assessing sample strength, the deviation from the linear elastic framework requires further scrutiny. As will be discussed in Sect. 4.3, the higher FIP can be attributed to the relatively small borehole size during pressurized injection [46].

**Fig. 10:**
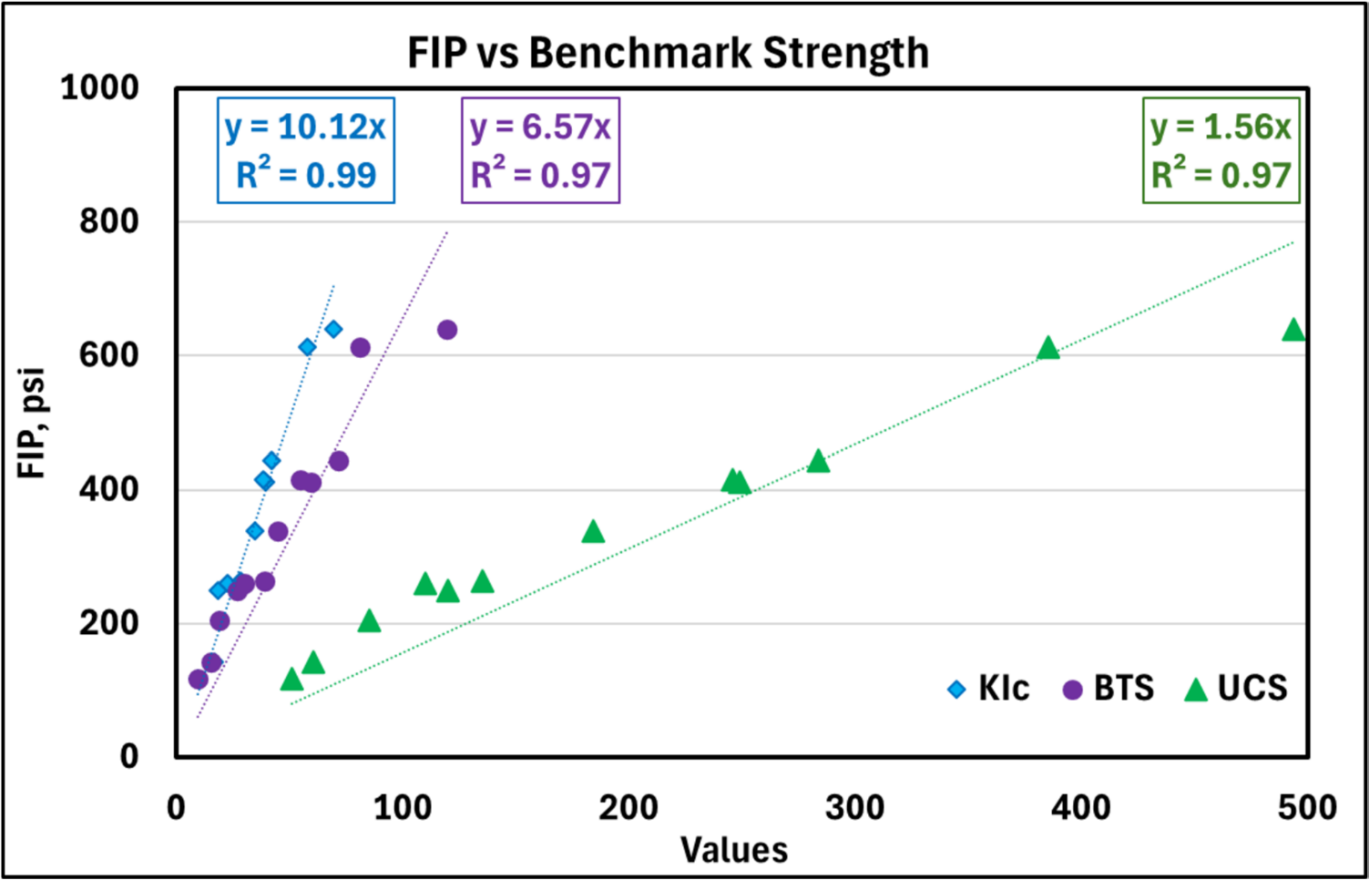
Relationship between mean fracture initiation pressure (in psi) and the mean benchmarked measures of strength: BTS (psi), UCS (psi) and K_Ic_ (psi√in)

The primary objective of this study is to determine whether FIP can be used as an effective measure of CPB strength. To validate its effectiveness, it is essential to assess whether FIP can distinguish between samples of varying strengths, similar to benchmarked strength (UCS, BTS, and K_Ic_) measurements. Figure 10 enables us to evaluate FIP's accuracy in relation to benchmark measures of strength. The data here is presented irrespective of the binder content and curing time point for the CPB samples, wherein a single point in the figure refers to the mean value of the given strength metric (abscissa value) and the corresponding FIP (ordinate value). The results indicate that, on average, the FIPs of the samples were 1.56 times, 6.57 times, and 10.12 times the UCS, BTS and K_Ic_, respectively. Additionally, the relationships between FIP-UCS, FIP-BTS and FIP-K_Ic_ are all linear in nature, with very high correlation coefficients (*R*^2^ exceeding 0.97). Notably, the regression models were fitted with an intercept at origin, as a sample with zero strength would ideally exhibit zero values for all strength measures.

To validate the statistical significance of the relationship between FIP and the strength measures shown in Fig. 10, *F*-tests were conducted. Pairwise analyses examined the relationships between FIP and each predictor: UCS, BTS, and KIC. The *F*-statistics for FIP-BTS, FIP-UCS, and FIP-KIC were 364.9, 387.8, and 1565.9, respectively, with all corresponding *P*-values below the 0.05 significance threshold, as seen in Table 4. These results confirm that FIP is a statistically significant predictor of BTS, UCS, and K_Ic_, and can be used as an effective measure of CPB strength.

**Table 4:** F-test results for FIP and geomechanical strength predictors

Predictor	Target	*F*-statistic	*P*-value	*P*-value < 0.05?	Interpretation
FIP	BTS	364.9	8.8e-10	Yes	Statistically significant
FIP	UCS	387.8	6.3e-10	Yes	Statistically significant
FIP	K_Ic_	1565.9	3.3e-13	Yes	Statistically significant

## Discussion

The primary focus of this section is to evaluate how effective the proposed fracturing technique is in measuring the strength of CPB. This assessment will be performed from an analytical and theoretical perspective based on the experimental data reported above.

### Electrical Conductivity-Based Measurements

Jafari et al. [28] introduced an empirical logarithmic model for EC-based strength assessment, using a regression equation of the form of Eq. 10. Here, $a$, $b$, and $c$ are fitting parameters.

10 $$UCS=a -b\times \mathit{ln}\left(EC+c\right)$$

**Fig. 11:**
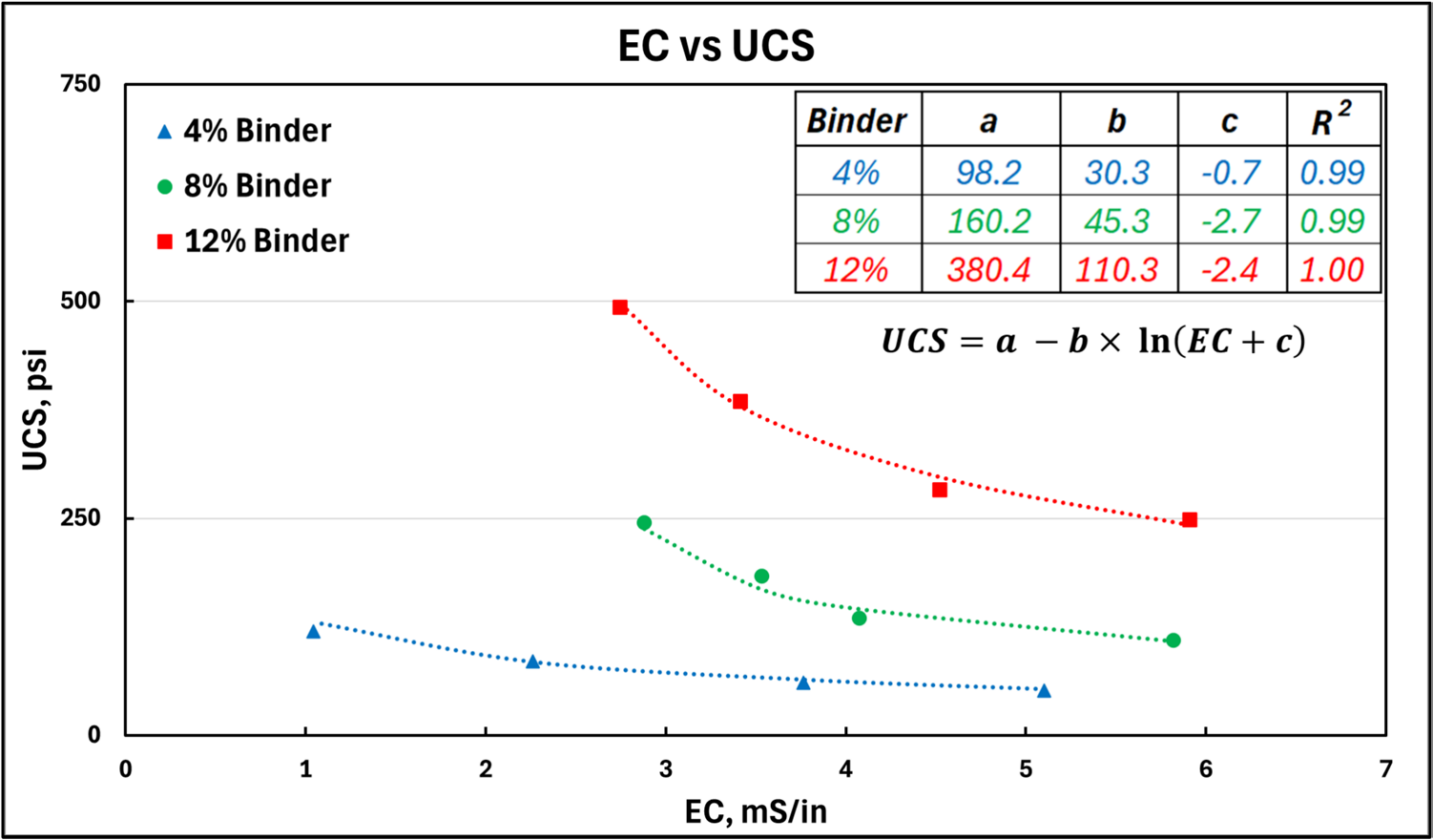
EC vs UCS of the CPB samples. The dotted line indicates the modeled results fit to the experimental data following the logarithmic regression model developed by Jafari et al. [28], with the corresponding fitting parameters and correlation coefficient presented in the insert table

Their study focused on early-age curing (1–7 days), while this work investigates late-stage strength (3–28 days). Regression fitting of the experimental data collected here, shown in Fig. 11, aligns with Jafari et al.’s model [28], but notable variations were observed in the fitting parameters (a, b). For 4%, 8%, and 12% binder samples, the values of a, b, and c were 98.2, 30.3, and − 0.7; 160.2, 45.3, and − 2.7; and 380.4, 110.3, and − 2.4, respectively, indicating that higher binder content, in particular, increases the value of a and b, consistent with Jafari et al.’s findings. Additionally, variation in the water-to-cement ratio also affected the values of the fitting parameters, although no definitive trend was observed.

A key observation made here is that a single fitting equation cannot be effectively used to define the EC-UCS relationship. This is especially true when there is a significant difference in the curing chemistry of different CPB samples. Additionally, the exact nature and the physical interpretation of the fitting parameters *a*,* b*, and *c* are not yet well understood. While the physical interpretation of the parameters remains unclear, EC monitoring offers promise for assessing early CPB strength, though its limitation is that previously unmonitored pastefill locations cannot be tracked. The hydraulic injection technique, as presented here, provides the ability to measure the strength of any CPB location without the need for installation of sensors and infrastructure prior to any planned pastefill. Additionally, as the fracturing technique relies on the creation of small tensile fractures, it provides a direct measure of strength.

### Failure Analysis—Standard Failure Models

The linear elastic and thick-walled pressure vessel-based failure theories (Eqs. 2, 7–9) suggest that FIP during hydraulic fracturing in unconfined samples is primarily influenced by the material's tensile strength and the borehole and samples geometry. Therefore, FIP measurements should theoretically allow for direct estimation of tensile strength, comparable to values from benchmark tests like the BTS test. However, the FIP values measured in this study were and reported in Table 3, on average, 6.57 times higher than the BTS, leading to a significant overestimation of tensile strength, regardless of the specific failure theory considered.

**Fig. 12:**
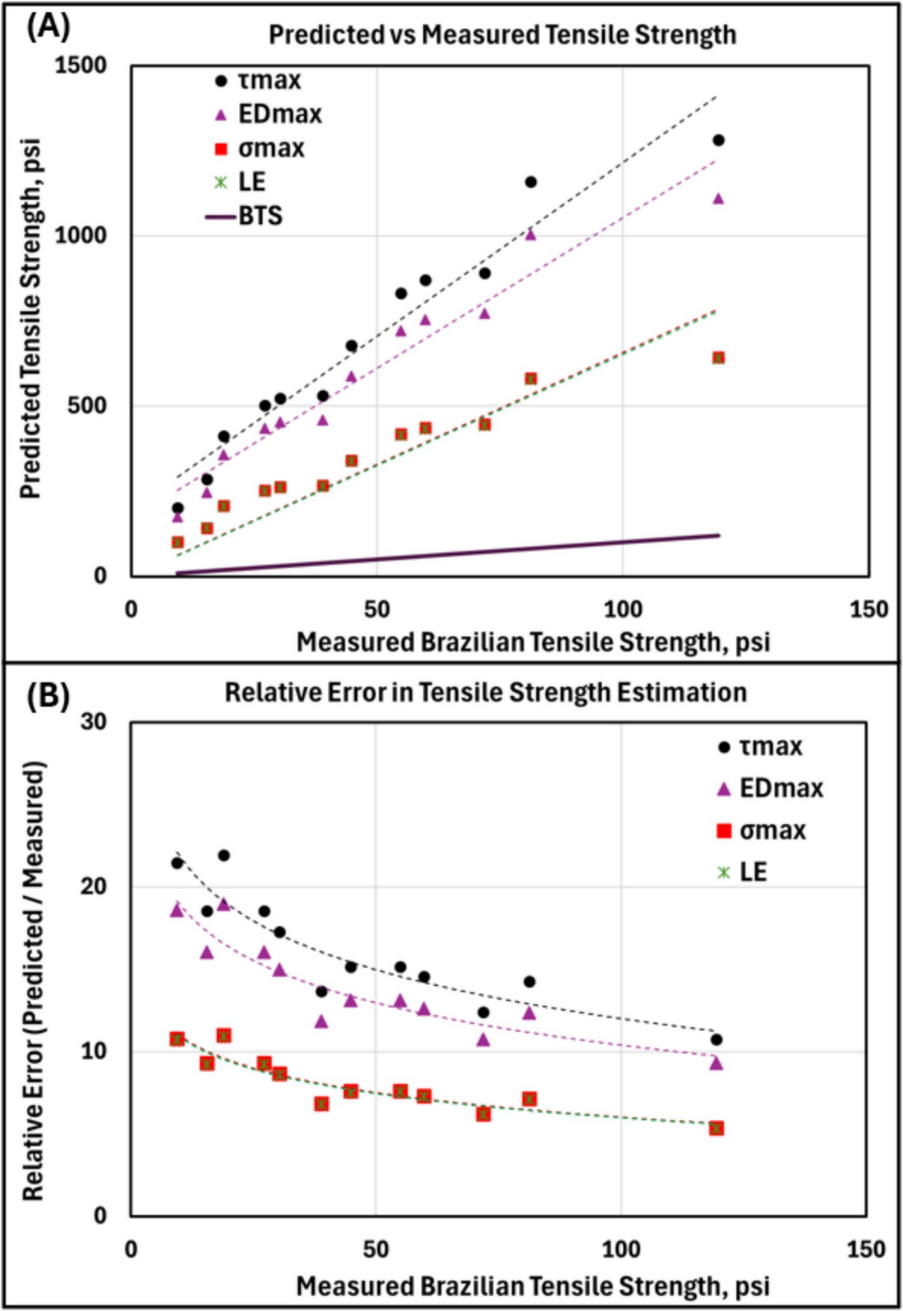
**A** Predicted tensile strength from hydraulic fracturing vs measured Brazilian tensile strength. **B** Relative error in the estimated values compared to the measured Brazilian tensile strength

Figure 12A compares the predicted tensile strength of CPB samples, derived from hydraulic fracturing data using various failure theories from Sect. 1.4, with the measured BTS. The failure theories include linear elastic (LE), maximum stress (${\sigma }_{\text{max}}$), maximum energy distortion (${ED}_{\text{max}}$), and maximum shear (${\tau }_{\text{max}}$) approaches. According to the LE theory, in unconfined tests, the expected tensile strength is equal to the FIP, represented by the green cross in Fig. 12A. Similarly, the ${\sigma }_{max}$ theory predicts strength values nearly identical to FIP due to the relatively small borehole size of 0.125″ diameter compared to the 2″ diameter core sample. The ${\tau }_{\text{max}}$ and the ${ED}_{\text{max}}$ theories predict even higher failure strengths.

As shown in Fig. 12B, the error in predicted tensile strength is most evident at lower tensile strength values. As the sample’s tensile strength increases, the prediction error decreases logarithmically. However, it remains significantly higher than the actual tensile strength. This highlights that standard failure theories are inadequate for estimating the tensile strength of cemented paste backfill based on FIP.

via a borehole, similar to the experimental setup reported here, will fail only when the average stress at a certain distance from the borehole wall exceeds the tensile strength of the intact material. This distance, referred to as the characteristic distance (*d*), is a material-specific property.

### Failure Analysis—Point Stress Model

Similar observations of fracture initiation pressures being much higher than the tensile strength of rocks—andesite, granite, and limestone were also reported by others [46–48]. The work reported here is the first to demonstrate this effect for CPB. This consistent trend was reasoned from the perspective of a Point Stress (PS) model [47]. The fundamental premise of the PS model is that, particularly for brittle materials, the inherent flaws within the material influence its response to hydraulic pressurization. When the borehole size is small, the material has a greater ability to redistribute stress, resulting in a higher apparent strength compared to samples pressurized with larger boreholes. Larger boreholes increase the likelihood of encountering significant flaws, leading to failure at pressures that accurately reflect the material’s true tensile strength. This concept led to the assumption that brittle materials, when subjected to internal pressurization. It is important to note that, while the failure mechanism in CPB also involves tensile failure at the borehole wall, CPB differs from crystalline rocks in its material characteristics. It is marked by high porosity, relatively low tensile strength, and bonding governed by cement hydration products such as CSH bonds. These features promote greater stress redistribution and flaw-controlled fracture behavior, amplifying the borehole size-dependent nature of fracture initiation observed in this study, as further explained below.

Following Ito and Hayashi’s work, the characteristic distance can be calculated once the fracture toughness and the material tensile strength is known [46]. The relationship is expressed as Eq. 11:

11 $$d= \frac{1}{2\pi }{\left(\frac{{K}_{Ic}}{{\sigma }_{T}}\right)}^{2}$$

Knowledge of the characteristic distance enables the direct calculation of either the fracture initiation pressure or the tensile strength of the material from the following relationships:

12 $$FIP= \frac{{\sigma }_{T}}{{I}^{2}}$$

13 $$I= \frac{{r}_{i}}{{r}_{i}+d}$$

**Fig. 13:**
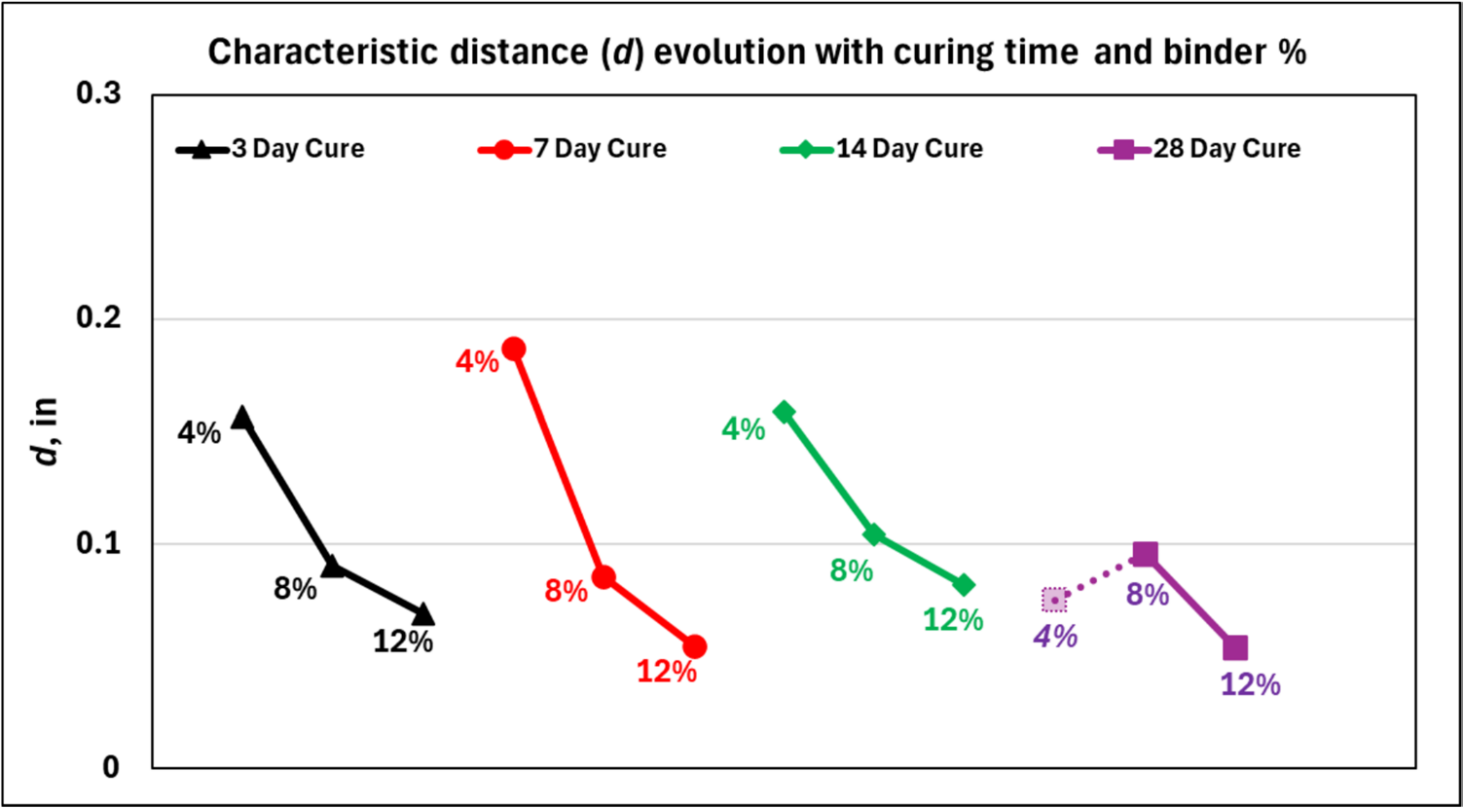
Evolution of characteristic distance in CPB samples as a function of curing time and binder percentage. Note: Since the K_Ic_ value for 4% CPB at 28-day cure was erroneous, the corresponding value for *d* is underpredicted

Measurement of the Brazilian tensile strength and K_Ic_, as reported in Table 3, enables calculation of the characteristic distance for the CPB samples. The calculated values for *d* are presented in Fig. 13. It should be noted that given the proportional dependence of *d* to the K_Ic_, the value obtained for 4% CPB at the 28-day cure time point is erroneous (see Table 3 Note), and should be regarded as an outlier. Disregarding the one outlier, for all CPB samples, the mean value of *d* was 0.1″ with a standard deviation of 0.04″. The data presented in Fig. 13 also suggests curing of CPB affects the value of *d* minimally. The mean and standard deviation of *d* for the 4% (not including the outlier), 8% and 12% CPB was 0.17″ (± 0.02″), 0.09″ (± 0.008″), and 0.06″ (± 0.01″), respectively. However, an increase in the binder content of CPB results in a decrease in the value of *d*. At this time, the exact reasoning to assess the dependence of characteristic distance on the binder content, and independence to the curing time is unknown, and should be the subject of future investigation.

**Fig. 14:**
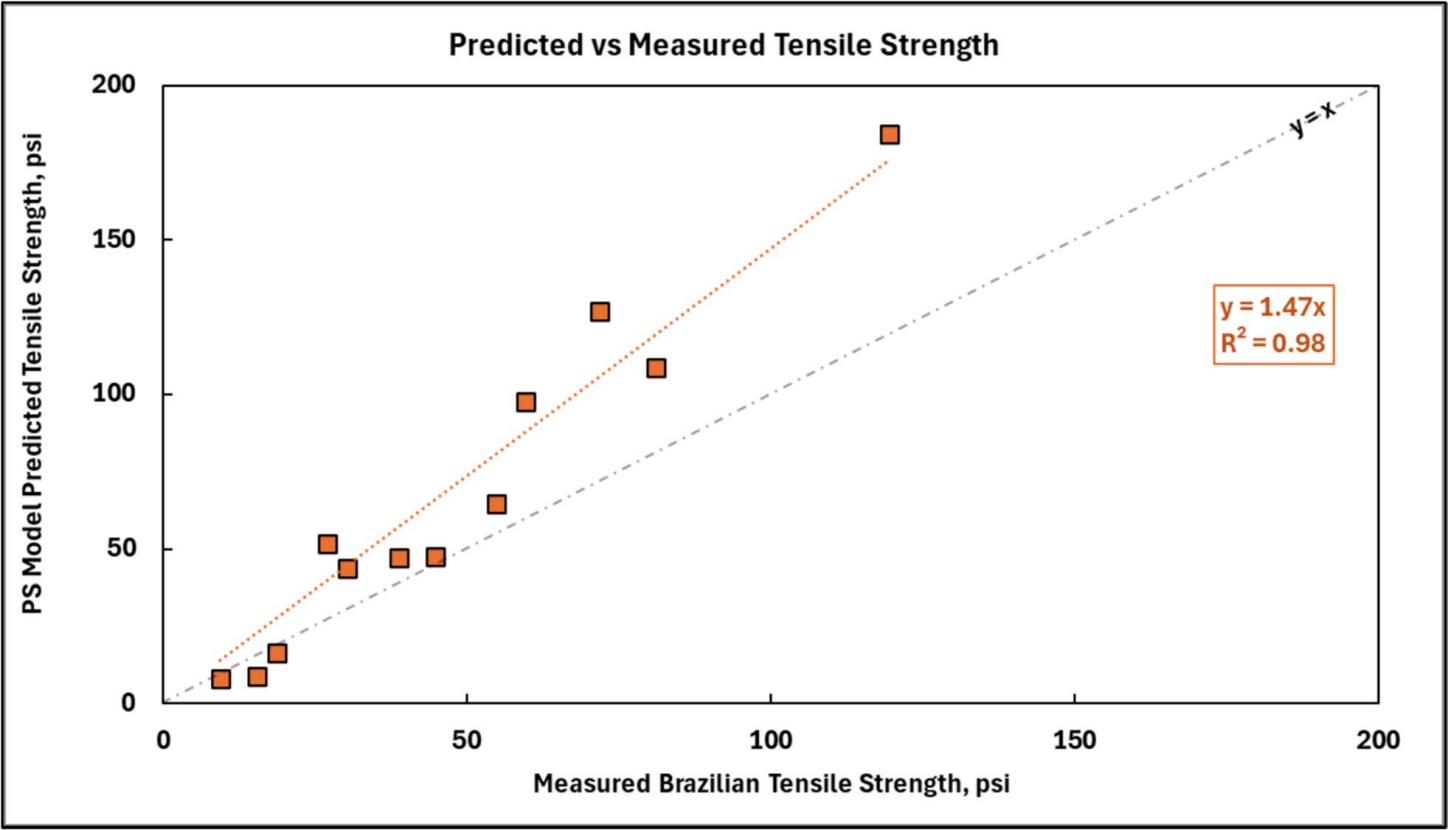
Predicted tensile strength from the Point Stress (PS) model versus measured Brazilian tensile strength (BTS) for CPB samples

After calculating the characteristic distance, *d*, it is possible to estimate the tensile strength of CPB using the measured mean values of FIP in accordance with Eqs. 12 and 13. Figure 14 below compares the tensile strength predicted by the Point Stress model with the measured Brazilian tensile strength of CPB. The orange squares in the figure, along with the linear regression fit, represent this data. As shown, the Point Stress model tends to overestimate the measured BTS, on average by a factor of 1.47.

**Fig. 15:**
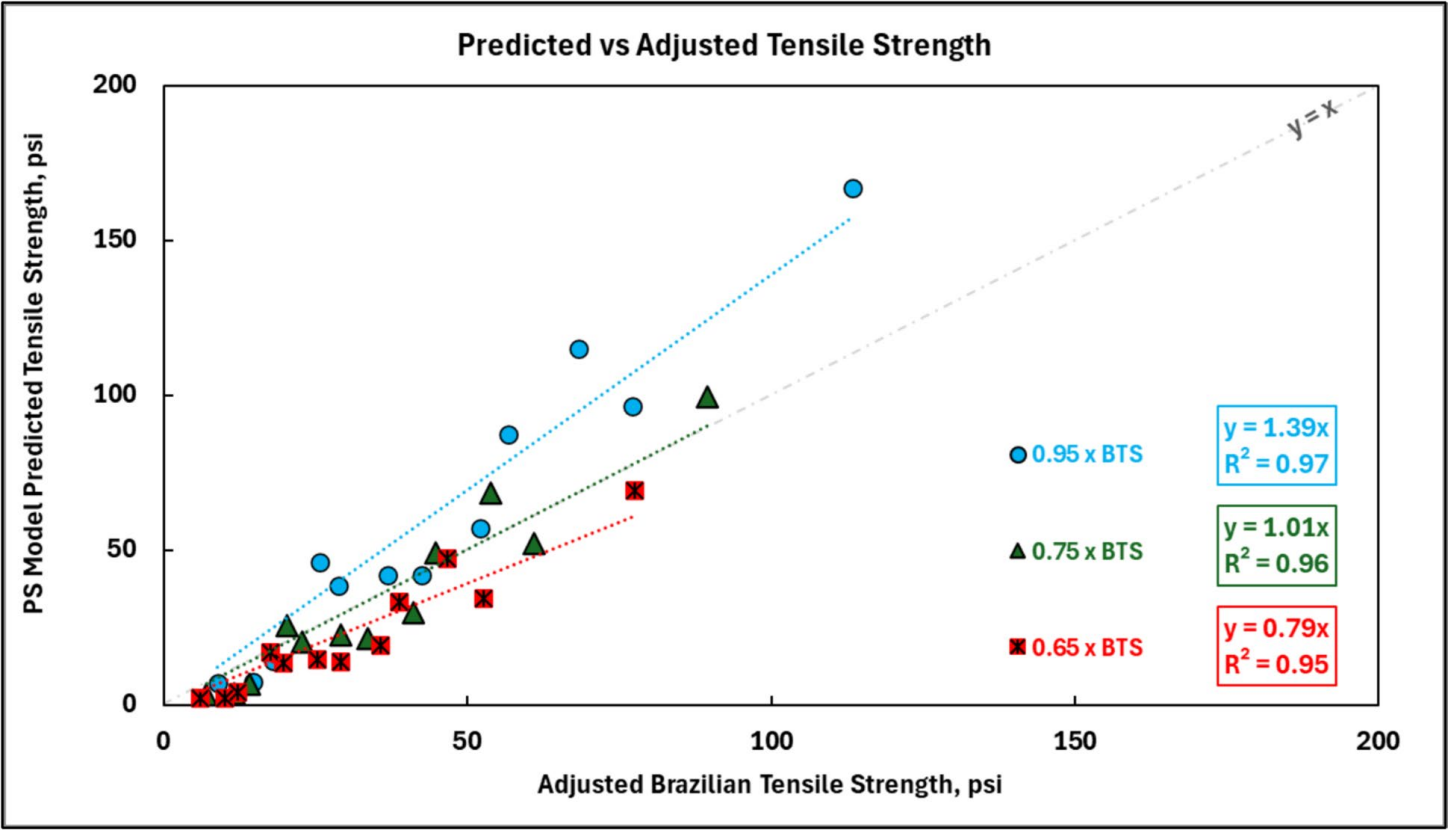
Predicted tensile strength from the Point Stress (PS) model versus adjusted Brazilian tensile strength (BTS) for CPB samples. Different scaling factors (0.95 × , 0.75 × , 0.65 × BTS) account for the range of overestimation of true tensile strength by BTS

It is important to note that the Brazilian Tensile Strength (BTS) is an indirect measure of tensile strength and tends to overestimate the true tensile strength of rocks and geomaterials. Studies have shown that the actual tensile strength for rocks and geomaterials is typically 68 to 93% of the measured BTS [49]. Specifically for cemented paste backfill, Seymour et al. reported a ratio of 0.71 between direct tensile strength and BTS for deslimed paste backfill from the Lucky Friday mine [25]. To address the overprediction of true tensile strength by the Brazilian tensile strength, the measured mean tensile strengths have been adjusted using factors of 0.95, 0.75, and 0.65. The predicted tensile strength using the Point Stress (PS) model was then re-evaluated. The results in Fig. 15 show that increasing the degree to which the BTS is reduced to estimate direct tensile strength reduces numerical value of the predicted tensile strength. For a factor of 0.75, represented by the green data points and regression plot, the PS model’s predicted tensile strength aligns closely with the adjusted tensile strength of the CPB samples. This result is interpreted to confirm that the reason for the discrepancy between predicted and observed tensile strengths is a consequence of the use of BTS as a direct representation of the direct tensile strength; the use of an appropriate factor to convert the BTS to a direct tensile strength, similar to the value reported by Seymour et al. [25], improves the accuracy of the Point Stress model in predicting the true tensile strength of cemented paste backfill, providing more reliable estimations for practical applications.

### Practical Implications

The validity of the PS model in interpreting the nature of hydraulic failure of CPB offers significant advantages for mining operations in evaluating the in situ strength of pastefill. Unlike current CPB strength testing methods, fracturing techniques can be readily implemented at operating mines, especially for in situ locations. Additionally, testing can also be conducted at plant facilities for calibration and characterization of CPB, using the straightforward experimental procedure described in this study. These advantages highlight the potential of hydraulic fracturing as a practical tool for CPB strength assessment.

Due to the relatively small borehole size used in this study, the FIP values provides a higher resolution of strength compared to traditional benchmark strength tests such as UCS, BTS, and K_Ic_. This high-resolution strength measurement can be particularly valuable in mining operations, where FIP obtained from small borehole diameters offers a reliable and precise assessment of CPB strength. It is to be noted that to enable use of FIP as a standardized strength measure at a mining operation, the correlations between FIP and the tensile/compressive strengths of CPB, for the various pastefill recipes commonly used at the operations will need to be established.

Directly measuring the tensile strength of CPB from FIP, particularly for small borehole radii, requires careful consideration of the size effect governed by the characteristic distance (*d*). This effect, detailed in Eqs. 12 and 13, emphasizes the dependence of FIP on borehole radius. Accurate estimation of *d* necessitates prior knowledge of both the tensile strength and fracture toughness of CPB, as outlined in Eq. 11, making direct tensile strength estimation from FIP less straightforward.

However, the PS model can still be leveraged effectively for direct tensile strength measurement. As indicated in Eq. 11, for sufficiently large borehole radii (${r}_{i} \gg d$), the value of $I$ approaches 1, and the measured FIP equates to the tensile strength of CPB. While this specific effect was not captured in the present study, prior research by Haimson and Zhao [48] on hydraulic fracturing of granite and limestone demonstrated that for borehole diameters exceeding 0.8″, the measured FIP aligns with the tensile strength of the material. Furthermore, the characteristic distance of a material, typically equal to only a few grain diameters [50], has been measured for CPB in this study as 0.1″ (± 0.04″). Based on these findings, borehole radii greater than 1″ would provide a reasonably accurate measurement of the tensile strength of CPB. However, before field-scale implementation, laboratory-scale assessments, similar to those reported in this work, are recommended to optimize borehole diameter design and ensure reliable application.

Additionally, from a cost–benefit perspective, the hydraulic fracturing method provides significant operational advantages, including direct in situ measurement capability, reduced sampling uncertainties, operational efficiency through decreased sample retrieval attempts, improved safety due to minimal sample handling, and enhanced measurement sensitivity evidenced by the strong linear correlations observed between FIP and benchmark strength measures (UCS, BTS, K_Ic_). However, potential costs such as specialized equipment (pressure transducers, hydraulic pump systems) and additional personnel training for adopting this method must be considered. A comprehensive techno-economic evaluation based on field-scale trials, accounting for equipment procurement, operational costs, labor, maintenance, and reduction in downtime relative to traditional methods, is recommended prior to commercial-scale implementation.

## Conclusion

The study introduces the concept of using controlled hydraulic fracturing as a method to directly measure the tensile strength of cemented paste backfill. Thus, offering a new practical approach compared to traditional tests (UCS, BTS and K_Ic_) which are used as traditional benchmark measures of CPB strength. This method is especially promising for application in in situ environments. The key conclusions drawn from this work are summarized below:

- The study validates the use of hydraulic fracturing to measure the strength of CPB, providing a practical alternative to traditional methods such as uniaxial compressive strength and tensile strength test.
- Fracture initiation pressure is shown to correlate strongly with CPB strength, and for the experimental conditions reported here, offering higher resolution in assessing strength compared to benchmark measures of CPB strength.
- The failure analysis suggests that standard linear elastic models do not accurately model the hydraulic fracturing of CPB. However, the point stress (PS) model can accurately model the fracture mechanics of CPB.
- The PS model indicates that FIP depends on borehole radius, with smaller radii yielding higher FIP values. This characteristic can be utilized to achieve higher resolution strength data, surpassing the accuracy of conventional direct and indirect tensile strength measurements. For direct tensile strength estimation using hydraulic fracturing, larger borehole radii can be employed, leveraging the validity of the PS model to ensure reliable results.

## Outstanding Knowledge Gaps and Future Work

The work presented here demonstrates the viability of using hydraulic fracturing to effectively characterize the strength of CPB. However, scaling this approach to a mine-wide application requires addressing several additional knowledge gaps:

- **Sensitivity to in situ stress**: Hydraulic fracturing applications are highly sensitive to the state of in situ stress. In deep mines, where significant post-mining closure occurs, it is essential to accurately assess the in situ stress to obtain a reliable measure of tensile strength. Therefore, the influence of varying confining stresses on fracture initiation pressure needs to be thoroughly evaluated.
- **Field-scale validation**: Field-scale studies are critical to validate the proposed technique. Many metal mining operations, such as those in the Carlin Trend, do not experience the excessive closure stresses typical of deeper mines. In these operations, the primary in situ stress acting on the backfill is often due to its own weight, which can be easily estimated. Implementing fracturing techniques in such mines would be relatively straightforward. Consequently, initial field-scale trials in mines with minimal closure stress would be the ideal next step in transitioning from laboratory to field-scale applicability.
- **Cost benefit analysis:** The work presented here validates the accuracy and reliability of hydraulic fracturing for CPB strength assessment. However, prior to commercial-scale implementation, a detailed techno-economic analysis based on operational-scale field trials is recommended. Future studies should quantify costs associated with equipment procurement, operation, labor, maintenance, and compare expected operational downtime reduction relative to traditional methods.
- **Characteristic distance of CPB**: The validity of the Point Stress model requires determining the characteristic distance for CPB samples, especially for small boreholes. However, the relationship between CPB’s characteristic distance and variables such as binder content, as well as the apparent independence to curing time, is not well understood. Further research is needed to investigate how factors like particle size, binder content, hydration chemistry, and curing time influence the characteristic distance in CPB.

## Data Availability

Data will be available on request.

## Nomenclatures

CPB: Cemented paste backfill
FIP: Fracture initiation pressure
UCS: Uniaxial compressive strength
BTS: Brazilian tensile strength
K_Ic_: Mode-1 fracture toughness
PS: Point stress (model)
HF: Hydraulic fill
CAF: Cemented aggregate fill
CRF: Cemented rock fill
WRF: Waste rock fill
EC: Electrical conductivity
CSH: Calcium silicate hydrate
LE: Linear elastic (model)
$${\sigma }_{T}$$: Tensile strength
${\sigma }_{1}$: Major principal stress
${\sigma }_{3}$: Minor principal stress
${P}_{0}$: Initial fluid pore pressure
*r*_*e*_: External thick-walled cylinder radius
*r*_*i*_: Borehole radius
*P*_*i*_: Internal fluid pressure applied to the borehole
$${\sigma }_{r}$$: Radial stress
$${\sigma }_{\theta }$$: Hoop stress
*A**, B*: Expression which are functions of the borehole geometry and fluid pressure
${\sigma }_{\text{max}}$: Maximum stress failure theory
${\tau }_{\text{max}}$: Maximum shear failure theory
${ED}_{\text{max}}$: Energy distortion failure theory
$a, b, c$: Regression fitting constants
$$d$$: Characteristic distance
$I$: Expression relating borehole radius to characteristic distance
